# Pre-Treatment Anemia Is a Poor Prognostic Factor in Soft Tissue Sarcoma Patients

**DOI:** 10.1371/journal.pone.0107297

**Published:** 2014-09-10

**Authors:** Joanna Szkandera, Armin Gerger, Bernadette Liegl-Atzwanger, Michael Stotz, Hellmut Samonigg, Ferdinand Ploner, Tatjana Stojakovic, Andreas Leithner, Martin Pichler

**Affiliations:** 1 Division of Clinical Oncology, Department of Medicine, Medical University of Graz, Graz, Austria; 2 Institute of Pathology, Medical University of Graz, Graz, Austria; 3 Clinical Institute of Medical and Chemical Laboratory Diagnostics, Medical University of Graz, Graz, Austria; 4 Department of Orthopaedic Surgery, Medical University of Graz, Graz, Austria; 5 Research Unit Genetic Epidemiology and Pharmacogenetics, Division of Clinical Oncology, Department of Medicine, Medical University of Graz, Graz, Austria; University of Louisville, United States of America

## Abstract

**Background:**

Anemia refers to low hemoglobin (Hb) levels, represents a common symptom and complication in cancer patients and was reported to negatively influence survival in patients with various malignancies. In the present study, we aimed to explore the prognostic impact of pre-operative Hb levels on clinical outcome in a large cohort of soft tissue sarcoma (STS) patients after curative surgery.

**Methods:**

Retrospective data from 367 STS patients, which were operated between 1998 and 2013, were included in the study. Cut-off levels for anemia were defined as Hb<13 g/dl in males and Hb<12 g/dl in females according to the current WHO guidelines. The impact of pre-operative Hb levels on cancer-specific survival (CSS) and overall survival (OS) was assessed using Kaplan-Meier curves. Additionally, Hb levels were compared for the prognostic influence on CSS and OS applying univariate and multivariate Cox proportional models.

**Results:**

Hb level was associated with established prognostic factors, including age, tumor grade, size and depth (*p*<0.05). Kaplan-Meier curves showed that low Hb levels were significantly associated with decreased CSS and OS in STS patients (*p*<0.001 for both endpoints, log-rank test). In multivariate analysis, we found an independent association between low Hb levels and poor CSS and OS (HR = 0.46, Cl 95% = 0.25–0.85, *p* = 0.012; HR = 0.34, Cl 95% = 0.23–0.51, *p*<0.001).

**Conclusion:**

The present data underline a negative prognostic impact of low pre-operative Hb levels on clinical outcome in STS patients. Thus, Hb levels may provide an additional and cost-effective tool to discriminate between STS patients that are at high risk of mortality.

## Introduction

Soft tissue sarcomas (STSs) are a relatively rare and heterogenous group of malignancies. They include a diverse group of non-epithelial extraskeletal tumors that show the histological differentiation of muscle, fat, nerves, and fibrous tissue. Their variable cellular appearance results in categorization of over 100 histologic sarcoma subtypes that differ in biology and behavior and make diagnosis and treatment challenging for clinicians [1]. Accordingly, the overall 5-year survival rate is only 50%, and especially STS patients that present with high grade tumors are at significant risk of disease recurrence [2]. The most important prognostic factors for local recurrence and distant metastasis are tumor size, tumor grade, histologic subtype, tumor depth and site and age at diagnosis [3]. However, predicting the outcome of STS patients remains sub-optimal and lacks predictive accuracy. Thus, there is an urgent need to identify additional prognostic markers. Blood-based biomarkers seem to be especially attractive for prognosis purposes, which might help clinicians to adopt preventive and therapeutic strategies for high risk patients [4]–[6].

Anemia is the most common hematologic abnormality in cancer patients. Although anemia incidence varies with types of malignancies and disease stage, it has been assumed that over 40% of all cancer patients are anemic at the time of diagnosis, a rate that increases up to 80% in patients with advanced disease [7]. The origin of cancer-related anemia is often multifactorial. It is a consequence of dysfunction of iron metabolism, inadequate production of erythropoietin (Epo) and inadequate response of the bone marrow to endogenous Epo, suppressed hematopoiesis through bone marrow infiltration, increased destruction of red blood cells, reduced number of erythroid progenitor cells in the bone marrow and production of inflammatory cytokines [8]–[10]. As the clinical symptoms of anemia start slowly, hemoglobin (Hb) level represents the most important predictor in guiding anemia evaluation and treatment. According to the World Health Organization (WHO), the cut-off levels for anemia were defined as Hb levels <13 g/dl in males and Hb levels <12 g/dl in females [11]. Recent studies indicate that a low Hb level is an unfavorable prognostic factor in diverse cancer types, including non-small cell lung cancer (NSCLC), ovarian carcinoma and pancreatic cancer [12]–[14]. To the best of our knowledge, only one similar sized study in surgical treated STS patients reported about a poor prognostic value of Hb levels [15]. Therefore, the present study was conducted to externally validate the prognostic significance of pre-operative Hb levels on cancer-specific survival (CSS) and overall survival (OS) in a large cohort of STS patients who underwent curative surgery.

## Materials and Methods

### Subjects

Three hundred and sixty seven patients with histologically confirmed STS who have been operated between March 1998 and August 2013 at the Department of Orthopaedic Surgery, Medical University of Graz, were retrospectively analyzed. Patients without available laboratory parameters or histological proven diagnosis were not included in this study. All patients were of Caucasians ancestry. The date of last follow-up documentation was the 1^st^ July, 2014. All patients were included in the follow-up program of the Department of Orthopaedic Surgery and the Division of Clinical Oncology, Medical University of Graz, providing follow-up examinations in regular intervals (3 month intervals in years 1–3, 6 months intervals in years 4–5, and 12 month intervals in years 6–15 after diagnosis). The laboratory data, including pre-operative Hb, was obtained by pre-operative determination one to three days before surgery was performed. Follow-up investigations included clinical check-up and radiological analyses (computed tomography alternating with X-ray of the chest, local magnetic resonance imaging, and abdominal ultrasound). Clinico-pathological data including histopathological diagnosis and tumor grade were retrospectively obtained from the patients history. For the present study, all histological specimens were centrally reviewed by an independent experienced soft tissue pathologist (B. LA). All sarcomas were diagnosed according to the current WHO classification of soft tissue and bone tumors [1]. Tumors were graded according to the French Federation of Cancer Centres Sarcoma Group (FNCLCC) grading system if possible or tumor grade was defined by tumor entity [16]. Cases formerly classified as malignant fibrous histiocytomas have been re-classified according to the current diagnostic criteria [1], [17]. This study has been approved by the Institutional Review Board (IRB) of the Medical University of Graz. Written informed consent was obtained from all participants.

### Statistical analysis

The primary endpoint of this study was CSS, which was calculated from the date of diagnosis to the date of cancer-related death. Secondary endpoint included OS which was defined as time between diagnosis and death of any cause. For anemia, the pre-published and most commonly used cut-off value of Hb level <13 g/dl in males and Hb level <12 g/dl in females was applied [12], [15]. The association between the Hb level and clinico-pathological parameters was evaluated by non-parametric tests (Mann-Whitney U and chi square test). Patients’ clinical endpoints were calculated using the Kaplan-Meier method and compared by the log rank test. Backward stepwise multivariate Cox proportion analysis was performed to determine the influence of age, gender, tumor size, grade and depth and Hb levels on CSS and OS. Hazard ratios (HRs) estimated from the Cox analysis were reported as relative risks with corresponding 95% confidence intervals (CIs). All statistical analyses were performed using the Statistical Package for Social Sciences version 20.0 (SPSS Inc., Chicago, IL, USA) or R program. A two-sided *p*<0.05 was considered statistically significant.

## Results

One hundred and eighty three male patients and 184 female patients with STS were included in the study. The median follow-up time was 37 months (interquartile range 14 to 88 months). Forty seven patients were lost to follow-up. The 367 patients were histologically classified as follows: 101 myxofibrosarcomas, 94 liposarcomas, 43 leiomyosarcomas, 29 synovial sarcoma, 13 malignant peripheral nerve sheath tumours (MPNSTs) and 87 other histological subtypes. The primary tumour site was localized at the upper extremities (n = 86), lower extremities (n = 219), thorax/trunk (n = 52), retroperitoneal/intraabdominal (n = 6) and head/neck (n = 4). The tumor depth was defined superficial in 120 patients and deep in 247 patients. All STS patients underwent surgery, and 43 (11.7%) were administered adjuvant chemotherapy. Two hundred twenty-five (61.3%) of the 367 sarcoma patients received adjuvant radiation therapy for the primary tumour site. The resection margins were determined as wide in 331 and marginal in 36 STS patients. Of the 367 STS patients, 70 (19.1%) developed metastatic disease, of which 62 (16.9%) died due to their advanced disease state. Thirty three patients (9%) presented with local recurrence. Overall 136 (37.1%) patients died of any cause by their most recent follow-up.

We validated the pre-published values of Hb level <13 g/dl in males and Hb level <12 g/dl in females as the cut-offs for the continuous Hb. Consequently, we separated the STS patients into two groups according to low Hb (<13 g/dl in males and <12 g/dl in females) levels or high Hb (≥13 g/dl in males and ≥12 g/dl in females) levels and tested the association between pre-operative Hb levels and other clinico-pathological factors. We found a statistically significant association between a low Hb level and older age, higher tumor grade, deep tumor location and larger tumor size (*p*<0.05), whereas no correlation with gender, tumor site and histologic subtype could be demonstrated (Table 1).

**Table 1:** The relation between clinico-pathological parameters and pre-operative Hb levels of patients with soft tissue sarcoma (n = 367).

Characteristics	Hb female <12 g/dlor male <13 g/dl	Hb female ≥12 g/dlor male ≥13 g/dl	*p*-value
**Age at diagnosis (yrs.)**			
<60	10	88	0.009
≥60	60	209	
Gender			
Female	34	150	0.771
Male	36	147	
**Tumor depth**			
Superficial	12	108	0.002
Deep	58	189	
**Tumor grade**			
G1+G2	10	130	<0.001
G3	60	167	
**Tumor size**			
<5 cm	7	86	<0.001
5–10 cm	27	124	
>10 cm	36	87	
**Tumor site**			
Upper extremity	14	72	0.729
Lower extremity	46	173	
Thoracic/trunk	9	43	
Retroabdominal/intraabdominal	1	5	
Head/neck	0	4	
**Histologic subtype**			
Liposarcoma	12	82	0.178
Myxofibrosarcoma	23	78	
Leiomyosarcoma	10	33	
Synovial sarcoma	2	27	
MPNST	2	11	
Other	21	66	

MPNST, malignant peripheral nerve sheath tumor.

**Figure 1:**
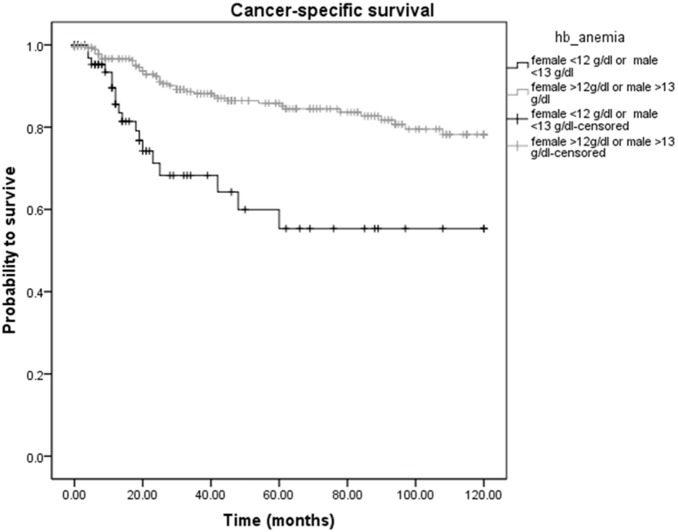
Kaplan-Meier curve for cancer-specific survival regarding high (≥13 g/dl in males and ≥12 g/dl in females) versus low (<13 g/dl in males and <12 g/dl in females) hemoglobin levels (*p*<0.001).

**Figure 2:**
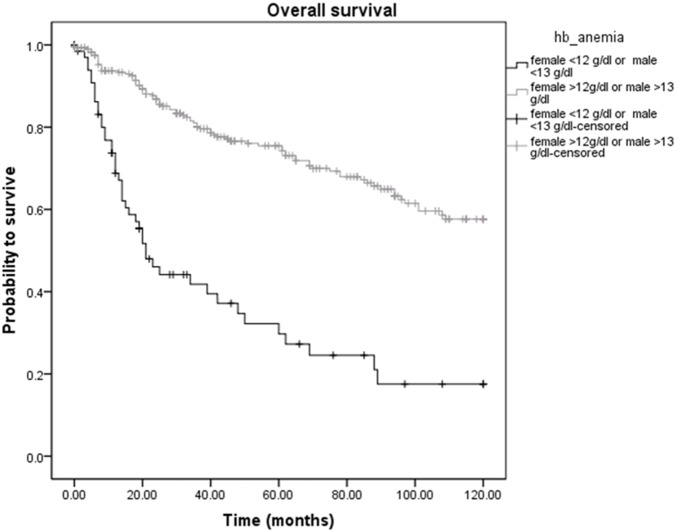
Kaplan-Meier curve for overall survival regarding high (≥13 g/dl in males and ≥12 g/dl in females) versus low (<13 g/dl in males and <12 g/dl in females) hemoglobin levels (p<0.001).

Analyzing the Hb levels into more detail, among the 367 STS patients, cancer-related death was diagnosed in 18 of 70 (25.7%) patients with low Hb levels and in 40 of 297 (13.5%) patients with high Hb levels (*p*<0.05). Overall deaths occurred in 44 (62.9%) patients with low Hb levels and in 82 (27.6%) patients with high Hb levels, respectively (*p*<0.05). Figures 1 and 2 show the Kaplan-Meier curves for CSS and OS and reveal that a low Hb level is a significant factor for decreased CSS and OS in STS patients (*p*<0.001 for CSS and OS, log-rank test).

To investigate whether serum Hb levels and other clinical-pathological factors are associated with clinical outcome of STS patients, univariate and multivariate Cox proportional models for CSS and OS were calculated. Univariate analysis identified a high tumor grade (G1+G2 versus G3, *p* = 0.002) and a low Hb level (<13 g/dl in males and <12 g/dl in females versus ≥13 g/dl in males and ≥12 g/dl in females, *p*<0.001) as poor prognostic factors for CSS in our study cohort (Table 2). For OS, we found a significant association between an older age (<60 versus ≥60, *p* = 0.002), a high tumor grade (G1+G2 versus G3, *p*<0.001) and a low Hb level (<13 g/dl in males and <12 g/dl in females versus ≥13 g/dl in males and ≥12 g/dl in females, *p*<0.001) and poor clinical outcome (Table 3). In the multivariate analysis that included age, gender, tumor grade, tumor depth, tumor size and the Hb level, we determined tumor grade and Hb level as independent prognostic factors for CSS (HR = 2.34, 95% CI = 1.28–4.30, *p* = 0.006; HR = 0.46, 95% CI = 0.25–0.85, *p* = 0.012; Table 2) and age, tumor grade and Hb level for OS (HR = 1.77, 95% CI = 1.10–2.85, *p* = 0.019; HR = 2.55, 95% CI = 1.67–3.89, p<0.001; HR = 0.34, 95% CI = 0.23–0.51, *p*<0.001; Table 3).

**Table 2:** Univariate and multivariate Cox proportional analysis regarding cancer-specific survival.

Parameter	Univariate analysis		Multivariate analysis	
	HR (95% Cl)	*p*-value	HR (95% Cl)	*p*-value
**Age at operation (yrs.)**				
<60	1 (referent)		1 (referent)	
≥60	1.98 (1.01–3.90)	0.048	1.75 (0.87–3.48)	0.115
**Gender**				
Female	1 (referent)		1 (referent)	
Male	0.92 (0.56–1.52)	0.757	0.78 (0.46–1.30)	0.335
**Tumor depth**				
Superficial	1 (referent)		1 (referent)	
Deep	1.36 (0.76–2.43)	0.301	1.22 (0.65–2.31)	0.538
**Tumor grade**				
G1+G2	1 (referent)		1 (referent)	
G3	2.45 (1.38–4.35)	0.002	2.34 (1.28–4.30)	0.006
**Tumor size**				
<5 cm	1 (referent)		1 (referent)	
5–10 cm	1.34 (0.96–1.87)	0.091	1.26 (0.86–1.84)	0.238
≥10 cm				
**Hb level**				
<13 g/dl in males and				
>12 g/dl in females	1 (referent)		1 (referent)	
≥13 g/dl in males and	0.33 (0.19–0.57)	<0.001	0.46 (0.25–0.85)	0.012
≥12 g/dl in females				

**Table 3:** Univariate and multivariate Cox proportional analysis regarding overall survival.

Parameter	Univariate analysis		Multivariate analysis	
	HR (95% Cl)	*p*-value	HR (95% Cl)	*p*-value
**Age at operation (yrs.)**				
<60	1 (referent)		1 (referent)	
≥60	2.08 (1.30–3.30)	0.002	1.77 (1.10–2.85)	0.019
**Gender**				
Female	1 (referent)		1 (referent)	
Male	0.94 (0.67–1.32)	0.718	0.83 (0.58–1.17)	0.281
**Tumor depth**				
Superficial	1 (referent)		1 (referent)	
Deep	1.09 (0.75–1.59)	0.648	0.99 (0.65–1.50)	0.956
**Tumor grade**				
G1+G2	1 (referent)		1 (referent)	
G3	2.92 (1.95–4.35)	<0.001	2.55 (1.67–3.89)	<0.001
**Tumor size**				
<5 cm	1 (referent)		1 (referent)	
5–10 cm	1.21 (0.96–1.51)	0.103	1.14 (0.88–1.47)	0.320
≥10 cm				
**Hb level**				
<13 g/dl in males and				
>12 g/dl in females	1 (referent)		1 (referent)	
≥13 g/dl in males and	0.26 (0.18–0.37)	<0.001	0.34 (0.23–0.51)	<0.001
≥12 g/dl in females				

Overall, in liposarcoma patients the incidence of anaemia was 12.8% (12 out of 94 patients) compared to 19% in the whole STS cohort. We analysed the incidence of anemia in these patients and found that the frequency of anaemia was significantly higher in patients with grade 2 (15%) or 3 (30.4%) liposarcomas compared to grade 1 (3.8%) liposarcoma patients (*p*  = 0.006).

## Discussion

In the present study, we found an independently significant association between low pre-operative Hb levels and poor clinical outcome in STS patients.

Pre-treatment anemia, indicated by low Hb levels, was reported to negatively influence clinical outcome in various types of cancer. Aoe et al reported a reduced median survival time (MST) in a large cohort of 611 NSCLC patients with anemia, defined as a Hb level <13 g/dl in males and <12 g/dl in females, at first presentation [12]. In pancreatic cancer, pre-operative Hb levels <12 g/dl were significantly associated with poor survival [13]. Another study, that included 206 patients with ovarian carcinoma, showed a decreased OS in patients with low Hb levels [14]. In STS, Nakamura et al demonstrated in 376 patients that low Hb levels (Hb levels <13 g/dl in men and <12 g/dl in women) correlated with established poor prognostic factors, including larger tumor size, higher tumor grade and older age. Furthermore, STS patients with anemia showed an independent association with reduced disease-specific survival (DSS) and event-free survival (EFS) [15]. In line with these findings, in the present study, low Hb levels were found predominantly in STS patients presenting with well-known factors associated with worse prognosis, such as high tumor grade, deep tumor location, large tumor size and older age. Similarly, we demonstrated that low Hb levels were significantly associated with decreased CSS in uni- and multivariate analysis. Additionally, an independent association between low Hb level and OS was observed in our study. These results might be explained by the fact that, on the one hand, in patients with malignancies, anemia might result from the extent of cancer-burden, and on the other hand may also be caused by co-morbidities that result in decreased survival, such as coagulation disorders, hemolysis, renal insufficiency, bleeding, or nutritional deficiencies [18]–[20]. Interestingly, in a subgroup analysis, Nakamura et al observed a higher incidence of anemia in patients with malignant fibrous histiocytomas (MFHs) and a low incidence of anemia in liposarcoma patients and attributed this to the fact that most of the liposarcoma patients presented with grade 1 liposarcomas. They also analyzed the relationship between anaemia, survival, and event separately for patients with malignant fibrous histiocytomas (MFHs) and liposarcomas and found that a low Hb level was a significant adverse prognostic factor for EFS in MFHs and liposarcomas. Moreover, patients with MFHs and anemia showed a significantly poorer DSS than those without anemia, whereas in liposarcomas, the rates of DSS did not significantly differ between those with and without anaemia [15]. To clarify these findings, in the present study, we analyzed the incidence of anemia in liposarcoma patients separately with respect to the grade and found that the frequency of anemia was higher in patients with grade 2 or 3 liposarcomas compared to grade 1 liposarcomas.

Based on recent data from experimental and clinical studies, there is increasing evidence that low Hb levels are associated with a poor tumor oxygenation and that up to 50–60% of locally advanced solid tumors may exhibit hypoxic tissue areas [21]. Hypoxia in solid tumors has been associated with malignant progression in terms of recurrence, loco-regional spread and distant metastasis, mediated by proteomic and genomic changes activating angiogenesis, anaerobic metabolism and other processes that enable tumor cells to survive or escape their oxygen-deficient environment, thus promoting the selection and expansion of more aggressive tumor clones [22]. Multivariate analyses have shown that hypoxia is a powerful prognostic factor in various cancer types, including STS. Brizel et al reported a significantly lower 18-month actuarial disease-free survival (DFS) rate in STS patients with tumor median oxygen pressure (pO2) values of <10 mmHg compared to patients with median pO2>10 mmHg (35% versus 70%, *p* = 0.01). Additionally, they demonstrated that the median pO2 for metastasized tumors was lower than for non-metastasized tumors [23]. In another study that investigated the relationship between tumor oxygenation and cell proliferation in STS, a significant association between the median pO2 and tumor cell potential doubling time was found, reporting the fastest proliferating tumor cells in the poorest oxygenated tumors [24]. Thus, we hypothesize that one reason for the poor clinical outcome in STS patients with low Hb levels in the present study might be due to tumor hypoxia. On the other hand, some cytokines, such as interleukin-6 (IL-6), have been demonstrated to induce anemia [25], [26]. It has been shown that IL-6 induces the liver to produce hepcidin. Hepcidin decreases iron absorption from the bowel and the gut and blocks iron utilization in the bone marrow, so that it is not available for erythropoiesis, resulting in cancer-related anemia [25]. Rutkowski et al reported that increased serum levels of IL-6 were found in the majority of STS patients included in their study [27].

In conclusion, the findings in our study suggest that low pre-operative Hb levels are significantly associated with decreased CSS and OS in STS patients after curative surgery. In context with the previously published study by Nakamura et al., we independently confirmed (or externally validated) their findings [15]. As both studies used a middle European cohort of Caucasian ancestry, evidence is supported that hemoglobin levels in STS patients are a general prognostic factor in other central European countries or Caucasians in the North American hemisphere. We identified anemia as an additional prognostic factor that might help to allow a more accurate strategy for stratifying patients with STS for risk of tumor-related and unrelated death. Markers like the haemoglobin can provide improvements in individual therapy modalities and follow-up schedules [4], [5]. In STS patients, administration of adjuvant chemotherapy is controversially discussed. Consequently, easily accessible, cheap and robust prognostic factors, such as Hb levels, might be helpful to allow treatment options to be tailored to the individual risk situation. With accurate prediction, patients at low risk for disease recurrence can be spared the toxicity of further treatment, whereas patients at high risk might be considered as candidates for additional adjuvant systemic therapy, novel experimental or more aggressive treatment approaches or more stringent follow-up schedules. As with all retrospective studies, our cohort is not without limitations. There have been several different surgeons involved over the years in the treatment of patients, and some other factors could also led to a selection bias, especially as we only included patients that were physically appropriate for a surgical procedure. In addition, we had no information about the influence of blood transfusions or administration of erythropoiesis stimulating agents on prognosis. Although the present investigations are limited due to their retrospective study design and the mixture of various histologic subtypes of STS, our data indicates that Hb levels, which are frequently measured in the routinely tested complete blood count panel, may represent a potentially important variable that can be included with other clinico-pathological parameters and laboratory findings to create new cancer prognosis assessment models.
